# Human Cytomegalovirus Encoded miR-US25-1-5p Attenuates CD147/EMMPRIN-Mediated Early Antiviral Response

**DOI:** 10.3390/v9120365

**Published:** 2017-12-01

**Authors:** Jun Chen, Sisi Xia, Xiangmin Yang, Huizi Chen, Fanni Li, Fenyong Liu, Zhinan Chen

**Affiliations:** 1 National Translational Science Center for Molecular Medicine, Xi’an 710032, China; cjun@whu.edu.cn (J.C.); yxiangmind@163.com (X.Y.); fannycpu@163.com (F.L.); 2 Cell Engineering Research Center & Department of Cell Biology, State Key Laboratory of Cancer Biology, Fourth Military Medical University, Xi’an 710032, China; 3 Key Laboratory of Combinatorial Biosynthesis and Drug Discovery, Ministry of Education, and School of Pharmaceutical Sciences, Wuhan University, Wuhan 430071, China; xiasisi@whu.edu.cn; 4 Department of Immunology, Zunyi Medical College, Guizhou 563000, China; chen_huizi@126.com; 5 Division of Infectious Diseases and Vaccinology, School of Public Health, University of California, Berkeley, CA 94720, USA; liu_fy@berkeley.edu

**Keywords:** human cytomegalovirus (HCMV), CD147/EMMPRIN, miR-US25-1-5p, cyclophilin A (CyPA), ERK/NF-κB signaling pathway, HCMV inflammatory disorders

## Abstract

Cellular receptor-mediated signaling pathways play critical roles during the initial immune response to Human Cytomegalovirus (HCMV) infection. However, the involvement of type-I transmembrane glycoprotein CD147/EMMPRIN (extracellular matrix metalloproteinase inducer) in the antiviral response to HCMV infection is still unknown. Here, we demonstrated the specific knockdown of CD147 significantly decreased HCMV-induced activation of NF-κB and Interferon-beta (IFN-β), which contribute to the cellular antiviral responses. Next, we confirmed that HCMV-encoded miR-US25-1-5p could target the 3′ UTR (Untranslated Region) of CD147 mRNA, and thus facilitate HCMV lytic propagation at a low multiplicity of infection (MOI). The expression and secretion of Cyclophilin A (sCyPA), as a ligand for CD147 and a proinflammatory cytokine, were up-regulated in response to HCMV stimuli. Finally, we confirmed that CD147 mediated HCMV-triggered antiviral signaling via the sCyPA-CD147-ERK (extracellular regulated protein kinases)/NF-κB axis signaling pathway. These findings reveal an important HCMV mechanism for evading antiviral innate immunity through its encoded microRNA by targeting transmembrane glycoprotein CD147, and a potential cause of HCMV inflammatory disorders due to the secretion of proinflammatory cytokine CyPA.

## 1. Introduction

Human cytomegalovirus (HCMV) is a widespread human β-herpesvirus with seroprevalence from 40 to 100%, which is closely related to socioeconomic and geographical conditions [1]. Life-long latent or persistent HCMV infection can be established in immunocompetent populations after the primary infection, and productive replication can occur from spontaneous reactivation due to immune abnormalities. Thus, HCMV is a major viral cause of mental retardation and sensorineural deafness in newborns, and particularly causes morbidity and mortality in transplant recipients and AIDS patients with an immunocompromised state [2]. Current studies also tie HCMV to multiple common chronic inflammatory diseases, such as cardiovascular disease, autoimmune diseases, inflammatory bowel diseases (IBDs), and certain cancers [3].

HCMV has a large linear dsDNA genome, with a size of 240-kbp encoding more than 150 open reading frames (ORFs) and 26 mature miRNAs encoded from 14 pre-miRNAs [4]. The large size of the viral genome has enabled co-evolution with its host for millions of years, to acquire various and even redundant strategies for immune evasion, to establish a life-long latent or persistent infection. As a result, a special balanced immune microenvironment for HCMV, from the interplay between the viral reactivation and immune response, was established, and intense research on its mechanisms could potentially contribute to a better understanding of the pathogenesis and biology of this virus.

Since most of the key immune cells, such as NK, T, and B cells, are not productively infected by HCMV, previous studies mainly focused on how the infection of permissive myeloid lineage or stromal cells alters the ability of these cells to prime and activate NK (natural killer cell) and T cells by down-modulating MHC (Major Histocompatibility Complex) and costimulatory molecules, suppression of activating signals, and provision of inhibitory molecules [5]. In fact, HCMV also uses diverse tactics to modulate the early immune response. Type I interferons (IFNs) and pro-inflammatory cytokines are rapidly expressed and secreted by infected cells upon the detection of HCMV binding and entry [6]. Gene expression profile assays have revealed that large numbers of antiviral genes, belonging to the interferon stimulated gene (ISG) family and the inflammatory cytokine family, are strongly induced by early events during HCMV entry [7]. Binding to cellular receptors by HCMV envelopeglycoproteins can initiate signal transduction pathways, and lead to the activation of NF-κB and interferon regulatory factor 3 (IRF3), which participates in the subsequent transcription of antiviral Type I IFNs and pro-inflammatory cytokines [8]. The early immune response is a pivotal time for the host to defend against HCMV infection. Recombinant IFN-α has been therapeutically used to control HCMV-induced retinitis in patients with AIDS [9] and congenital viremia with HCMV infection [10]. HCMV proteins have been shown to target early antiviral immune responses. For example, IE86, encoded by the HCMV immediate-early 2 (IE2) gene, can efficiently suppress HCMV or Sendai virus (Sev)-induced expression of IFN-β [11]. The HCMV tegument protein UL82 has been reported to inhibit Stimulator of Interferon Genes (STING)-mediated signaling for evading antiviral innate immunity [12].

Cellular receptor mediated signaling pathways are generally assumed to play an important role during the earliest immune response to virus infection. In previous studies, Toll-like receptor 2 (TLR2) has been reported to be critical in the early response to HCMV infection by recognizing the viral surface glycoproteins gB and gH [13]. Here, CD147/EMMPRIN, a type-I transmembrane glycoprotein of the immunoglobulin superfamily [14], was found and confirmed to play an important role in HCMV-triggered early antiviral signaling for the first time. Interestingly, we also found that CD147 expression was down-regulated at the late stages of HCMV infection, and that CD147 was a target gene for HCMV-encoded miR-US25-1-5p. Simultaneously, the expression and secretion of Cyclophilin A (sCyPA), which has been reported as a ligand for CD147 [15,16], were up-regulated in response to HCMV infection. CD147 is a multifunctional transmembrane glycoprotein that is involved in neural function, pro-inflammation, tumor invasion, and metastasis [17]. Extracellular CypA has been reported to induce the production of proinflammatory cytokines through direct binding with the ectodomain of CD147, as seen in our previous work [18]. The specific biological significance and mechanism of HCMV miR-US25-1-5p by targeting CD147/EMMPRIN, during the early antiviral response to HCMV infection, were further investigated and discussed in this study.

## 2. Materials and Methods

### 2.1. Cells and Viruses

Human foreskin fibroblasts (HFF cells, passages 9 to 14), Human astrocytoma U251 cells (U251 cells), and Human Embryonic Kidney 293 cells (293 cells) were maintained in Dulbecco’s modified Eagle medium (DMEM), containing 10% fetal bovine serum, as previously described [19,20]. Herpes simplex virus 1 (HSV-1) was provided by Fenyong Liu (University of California, Berkeley, CA, USA). To generate the U251-CD147 overexpressing (U251-CD147) and U251-Control (U251-C) cell lines, U251 MG cells were transfected with the pcDNA3.1-CD147 plasmid or the pcDNA3.1(+) (Invitrogen, Carlsbad, CA, USA) empty vector control plasmid using Lipofectamine^TM^ 2000, as prescribed by the manufacturer (Life Technologies, Waltham, MA, USA). After a 48-h incubation, transfected cells were selected for resistance in cell culture medium containing 600 μg/mL G418 (Life Technologies). After two to three weeks of selection, single clones were randomly isolated, and each was plated separately. The CD147 levels in individual selected clones were determined by Western blot analysis. The maintenance medium for cell lines was supplemented with 200 μg/mL G418. The bacterial artificial chromosome (BAC)-derived clinical strain NR-1 with enhanced green fluorescent protein (eGFP) expression cassette, provided by Dr. Ke Zen (Nanjing University, Nanjing, China), was used in this study [21]. HCMV mutant (mUS25-1-5p) and the revertant wild type (RvWT) was generated using a bacterial recombineering method, as previously described [22]. In brief, approximately 300 bp of the miR-US25-1 flanking region was cloned into the pcDNA3.1(+) vector. The three nucleotides in the seed region of miR-US25-1-5p were mutated using site-directed polymerase chain reaction (PCR). Then, the PCR-amplified products containing point mutations of miR-US25-1-5p were used in the second round of recombination to replace the Tet/Str cassette that inserted in the region of miR-US25-1-5p at first. The amplified DNA fragments were transformed into *E. coli* EL350 cells by electroporation (Bio-Rad, Hercules, CA, USA). The bacteria harboring the rescued BACs were selected in the presence of streptomycin. Mutants were confirmed using BAC DNA sequencing. Similarly, the BAC of RvWT was generated based on the newly constructed mUS25-1-5p, with two rounds of recombination. HCMV WT, mUS25-1-5p, and RvWT were propagated in HFF cells, and virus stocks were stored in DMEM supplemented with 10% fetal bovine serum (FBS) and 1.5% bovine serum albumin (BSA) at −80 °C.

### 2.2. Reagents and Antibodies

Cyclosporin A (CsA) reagent was obtained from Sigma-Aldrich (St. Louis, MO, USA). CD147 antibodies (HAb 18, IgG1) were prepared in our laboratory [23]. Dylight 594-conjugated secondary antibody, used for immunofluorescence, was from Life Technology (San Jose, CA, USA). We also used anti-HCMV IE1/2 mouse mAb (ab53495, Abcam, Cambridge, UK), rabbit anti-human CyPA mAb (ab3563, Abcam), phospho-MEK1/2 (Ser217/221) rabbit mAb (#9154, Cell Signaling Technology (CST), Danvers, MA, USA), Erk1/2 rabbit mAb (#4695, CST), IRF-3 rabbit mAb (#11904, CST), phospho-IRF-3 (Ser396) rabbit mAb (#29047, CST), NF-κB p65 rabbit mAb (#8242, CST), phospho-NF-κB p65 (Ser536) rabbit mAb (#3033, CST), and anti-GAPDH mouse mAb (60004-1-Ig, Proteintech, Rosemont, IL, USA).

### 2.3. Construction of Plasmids 

The following plasmids were used: CD147 pLKO.1 lentiviral shRNA (A6) and non-target shRNA control plasmid (pLKO.1-NTC) were purchased from Open Biosystems (GE Healthcare, Little Chalfont, UK). HCMV-encoded miR-US25-1-5p pLKO.1 lentiviral shRNA (pLKO.1-US25-1-5p) was constructed in this study. pcDNA3.1(+) empty vector was obtained from Invitrogen (Carlsbad, CA, USA). Full-length CD147-expressing plasmid pcDNA3.1-CD147 was constructed in our laboratory [24]. Then, the extracellular domain (residues 1–185 of CD147) or intracellular domain (residues 230–269 of CD147) were deleted to generate pcDNA3.1-CD147-dECD and pcDNA3.1-CD147-dICD, respectively. The NF-κB-response promoter reporter plasmid (pNF-κB-Luc) and IFN-β promoter reporter plasmid (pIFN-β-Luc) were obtained from Beyotime (Shanghai, China). Dual luciferase miRNA target expression vector (pmirGLO) and the Renilla luciferase control reporter plasmid (pRL-TK) were purchased from Promega (Madison, WI, USA). The pmirGLO-CD147^3′UTR^ plasmid was made by inserting the 3′ UTR of the human CD147 gene into the pmirGLO empty vector using the primers as follows: (forward) 5′-AAGCTAGCGGCAGGTGGCCCGAGGACGCTCCCTG-3′ and (reverse) 5′-AGTCTAGAGAGGGTGGAGGTGGGGGCGATC-3′. Site-directed mutagenesis was performed using a QuikChange Lightning Multi Site-Directed Mutagenesis Kit (Stratagene, San Diego, CA, USA) on the pmirGLO-CD147^3′UTR^ to generate a pmirGLO-CD147^3′UTRm^ plasmid with the following primers: (forward) 5′-AGTCATGGCCGGGTAGACAGCACAGCCTTCT-3′ and (reverse) 5′-AGAAGGCTGTGCTGTCTACCCGGCCATGACT-3′.

### 2.4. Indirect Immunofluorescence Assay (IFA)

Cells that were grown on chambered cover slips were infected with HCMV strain NR-1 at a multiplicity of infection (MOI) of 5. At 6 h posttransfection, cells were fixed with 4% formaldehyde and blocked with 4% bovine serum albumin (BSA) in PBS and stained with primary mouse IE1/2 antibody (ab53495, Abcam, Cambridge, UK), and then incubated with the secondary antibody Dylight 594 anti-mouse IgG (Life Technology). Cell nucleus was stained with 4′,6-diamidino-2-phenylindole (DAPI) (Invitrogen). Images were captured with a Nikon Eclipse TE300 microscope (Diagnostic Instruments, Inc., Sterling Heights, MI, USA) [20]. The digital images were subsequently merged using FV10 ASW V4.1 software (Olympus, Tokyo, Japan).

### 2.5. RNAi-Transduced Stable Cells

The 293 cells were co-transfected with the two packaging plasmids (psPAX2 and pMD2G), together with a control or RNAi pLKO.1 lentiviral plasmid using Lipofectamine^TM^ 2000 (Invitrogen). The sequence for CD147 shRNA was: 5′-CCCATCATACACTTCCTTCTT-3′ (siCD147); the sequence for HCMV-miR-US25-1-5p shRNA was: 5′-CCGCTCAGTGGCTCGGACC-3′ (miR-US25-1-5p). After 24 h, cells were incubated with fresh medium without antibiotics for another 24 h. The medium containing the recombinant virus was collected and filtered, and then added to HFF or U251 cells in the presence of 6 mg/mL polybrene. The infected cells were selected by adding puromycin (4–6 mg/mL) to the culture medium for 14 days before additional experiments. The silencing of expression was verified by qPCR and Western blot.

### 2.6. Reporter Gene Assays

Cells (1 × 10^5^) were seeded on 12-well plates and the following day were transfected using LipofectamineTM 2000 (Invitrogen) and the indicated plasmids. For transfection efficiency normalization, 0.01 μg Renilla luciferase reporter plasmids (pRL-TK) were added to each transfection. The total amount of transfeced DNA was maintained at a consistent level by adding empty vector DNA. Then, 36-h post-transfection, cells were lysed with 200 μL passive lysis buffer (Promega). Supernatants clarified by centrifugation were used to perform Luciferase assays using the dual luciferase assay kit (Promega). The values of Firefly luciferase activities were normalized to Renilla luciferase activities. All of the reporter assays were repeated at least three times in triplicate. Data shown are average values ± standard deviation (SD) from one representative experiment.

### 2.7. Real-Time PCR Quantitation of HCMV DNA Level

Viral DNA was extracted from mock or HCMV-infected cells using a DNeasy tissue kit (Qiagen, Hilden, Germany). Intracellular viral DNAs were quantified using primers and a probe for amplifying the HCMV UL83 DNA region, as previously described [19]. The reaction was performed in a 20-μL amplification mixture (2 μL of DNA extract, 10 μL of 2 × Premix Ex Taq^TM^ (TaKaRa, Dalian, China), 0.4 μL each of primer at 10 μM, 0.8 μL of the fluorogenic probe at 5 μM, 0.4 μL 50 × ROX Reference Dye II) using an ABI 7500 device (Applied Biosystems Inc., Foster City, CA, USA). PCR cycling conditions were as follows: 95 °C for 30 s, 45 cycles at 95 °C for 5 s and 60 °C for 34 s. Viral genome copies were normalized to cellular RNase P with thepreviously described primers and probe [25]. Unknown sample values were determined with a standard curve of known copy numbers of UL83 (NR-1 BAC) and RNase P. Each set of assays was repeated three times in triplicate, and data that are shown are average values ± SD from one representative experiment.

### 2.8. RNA Extraction and RT-qPCR Assays

Total RNA was isolated with TRIzol reagent (Invitrogen) and cDNA was reverse transcribed using a PrimeScriptTM RT Reagent Kit (TaKaRa). SYBR-Green real-time PCR (RT-PCR) was performed with the ABI 7500 device (Applied Biosystems Inc.) using SYBR Premix Ex Taq II (2×) (TaKaRa). Cellular GAPDH (Glyceraldehyde-3-phosphate dehydrogenase) was used to normalize the RNA inputs. Relative changes in gene expression were analyzed using the 2^−ΔΔCt^ method. All primers were synthesized by Shanghai GenePharma Co., Ltd., and are listed as following:

CD147: (forward) 5′-TCGCGCTGCTGGGCACC-3′ and (reverse) 5′-TGGCGCTGTCATTCAAGGA-3′; Cyclophilin A: (forward) 5′-CATGGTCAACCCCACGTGTTCTT-3′ and (reverse) 5′-TAGATGGACTTGCCACCAGTGCCAT-3′; IFNB1: (forward) 5′-AGGACAGGATGAACTTTGAC-3′ and (reverse) 5′-TGATAGACATTAGCCAGGAG-3′; IL-6: (forward) 5′-GACAGCCACTCACCTCTTCA-3′ and (reverse) 5′-AGTGCCTCTTTGCTGCTTTC-3′; ISG15: (forward) 5′-CACAGCCATGGGCTGGGACCTG-3′ and (reverse) 5′-GCACGCCGATCTTCTGGGTGA-3′; TNF-α: (forward) 5′-ATGAGCACTGAAAGCATGATCCGG-3′ and (reverse) 5′-CTACAACATGGGCTACAGGCTTGT-3′; GAPDH: (forward) 5′-AGCAATGCCTCCTGCACCACCAAC-3′ and (reverse) 5′-CCGGAGGGGCCATCCACAGTCT-3′.

### 2.9. Transfection of siRNA into Cells

The chemically synthetic siRNA, targeting the CD147 mRNA CDS region (miR-CD147, 5′-GUUCUUCGUGAGUUCCUCtt-3′) [26], HCMV miR-US25-1-5p (MirBase, MIMAT0001581), miR-US25-1-3p (MirBase, MIMAT0004755), and 13 other screened HCMV-encoded mature microRNAs (http://www.mirbase.org/) were synthesized by Ribobio Co., Ltd. (Guangzhou, China), along with the silencer negative control siRNA (C-siRNA) molecules. The siRNAs were transfected into cells using Lipofectamine^TM^ 2000 (Invitrogen), according to the manufacturer’s instructions.

### 2.10. Western Blot Analysis

Cells (1 × 10^8^) were washed with cold PBS and lysed with 1 mL NP40 lysis buffer with 1 mM β-mercaptoethanol and protease inhibitor Cocktail (Roche, Basel, Switzerland) for 30 min on ice. Cell lysates were centrifuged at 12,000 rpm for 30 min, and supernatants were collected. The protein concentration was determined by BCA assay. Proteins were separated by 8% SDS-polyacrylamide gel electrophoresis and transferred to polyvinylidene fluoride (PVDF) membrane (GE Healthcare). Block in 5% BSA was performed for 1 h in TBS with 0.1% Triton X-100 (TBST), followed by incubation with primary antibody solution overnight at 4 °C. Then, the membrane was rinsed 3–5 times for 5 min with TBST. After rinsing, the membrane was incubated with the horse radish peroxidase (HRP)-conjugated secondary antibody solution for 2 h at room temperature. After another rinsing, the membrane was subsequently reacted with the chemiluminescent substrate, according to the recommendation of the ECL Western blot detection reagent kits (Thermo Scientific, Waltham, MA, USA), and were quantitated chemiluminescent signals using a CCD camera-based imager (Bio-Rad, Hercules, CA, USA). Quantitation was performed in the linear range of protein detection and analyzed by Image Lab 4.0 software (Bio-Rad). The experiments were repeated at least three times in triplicate, and a representative result is shown.

### 2.11. Viral Infection and Growth Curves

Cells (1 × 10^6^) were either mock or HCMV infected at a MOI of 0.05 to 5 in 1.5 mL DMEM with 1% fetal bovine serum. The medium was replaced with 10% fetal bovine serum containing DMEM after 2 h of incubation. Protein extracts prepared from the infected cells at 3 to 12 days post-infection, were analyzed by Western blot analysis. To determine viral growth level, cells (1 × 10^5^) were infected with HCMV at an MOI of 0.05 or 3. Viral stocks were prepared with the total cells and the medium was harvested 3 to 11 days post-infection by adding an equal volume of 10% (*w*/*v*) skim milk, followed by ultrasonication. The titers of the viral stocks were determined in 1 × 10^5^ HFF cells, with standard virus plaque assay and the number of plaques were counted 10 to 14 days after viral infection. The values obtained were averages ± SD from triplicate experiments [19].

### 2.12. Statistical Analysis

All of the statistical analyses were performed with GraphPad Prism version 6.00 (GraphPad Software, San Diego, CA, USA). Experiments were repeated at least three times in triplicate. Data are expressed as mean ± standard deviation. Means between two groups were compared by using unpaired *t*-test or one-way analysis of variance (ANOVA) with post hoc Bonferroni *t*-test. Differences were considered statistically significant at *p* < 0.05.

## 3. Results

### 3.1. Involvement of CD147 in HCMV Triggered Early Antiviral Response

**Figure 1:**
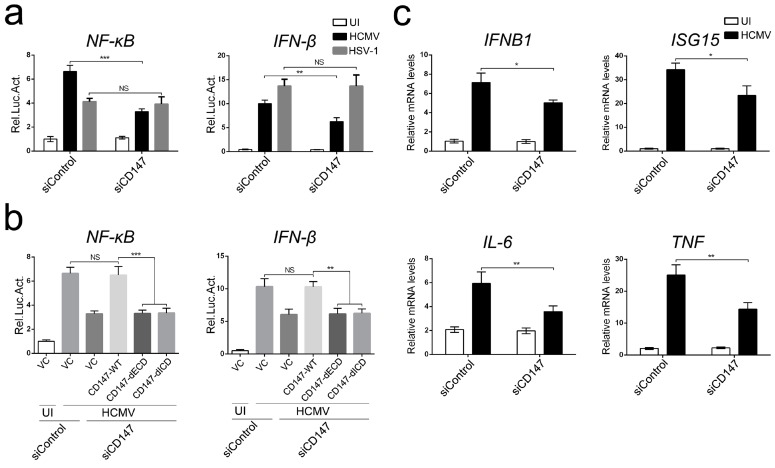
CD147 is involved in Human Cytomegalovirus (HCMV)-triggered early antiviral response. (**a**) Luciferase assay analyzing NF-κB and IFN-β promoter activity in non-targeting control siRNA (siControl) or CD147-specific short hairpin RNA (siCD147) stable U251 cells (2 × 10^5^) transfected for 36 h with a plasmid encoding an NF-κB or IFN-β firefly luciferase reporter (0.1 μg), along with a Renilla luciferase reporter control plasmid (pRL-TK, 0.01 μg), then left uninfected (UI) or infected for 6 h with HCMV or HSV-1 (multiplicity of infection (MOI) = 0.5); (**b**) Luciferase assay analyzing NF-κB and IFN-β promoter activity in siControl or siCD147 stable U251 cells (2 × 10^5^) transfected for 36 h with a plasmid encoding an NF-κB or IFN-β firefly luciferase reporter (0.1 μg) and an expression plasmid for the wildtype CD147 (CD147-WT), or the mutants (CD147-dECD and CD147-dICD) (0.1 μg), along with a Renilla luciferase reporter control plasmid (pRL-TK, 0.01 μg), then left uninfected (UI) or infected for 6 h with HCMV before the luciferase assays were performed (MOI = 0.5); and, (**c**) Quantitative real-time polymerase chain reaction (RT-PCR) analysis of *IFNB1*, *ISG15*, *IL-6* and *TNF* mRNA in siControl or siCD147 stable human foreskin fibroblast (HFF) cells with HCMV uninfected (UI) or infected (MOI = 0.5) for 6 h. The graphical data are presented as the means ± the SDs (*n* = 3); NS denotes not significant (*p* > 0.05); * denotes *p* < 0.05, ** denotes *p* < 0.01 and *** denotes *p* < 0.001.

Many transmembrane receptor proteins have been proposed to mediate signal transduction pathways that activate the early antiviral immune to HCMV entry [27]. Here, we have investigated the role of CD147/EMMPRIN in this response. We established two stable knockdowns of CD147, with a CD147-specific short hairpin RNAs (siCD147) targeting the 3′ UTR of cellular CD147 mRNA, and two corresponding non-specific RNAi stable cells as controls (siControl) via lentiviral-based transduction of Human astrocytoma U251 cells (U251 cells) and Human foreskin fibroblasts cells (HFF cells). CD147 expression was markedly reduced in siCD147-transduced cells when compared to the level in siControl cells, as observed with immunoblotting (Figure S1). Then, we measured the effect of CD147 knockdown on HCMV-induced activation of the IFN-β and NF-κB promoters using a dual-luciferase reporter assay in U251 cells. CD147 knockdown significantly reduced the activation of NF-κB and IFN-β promoters that were stimulated by infection with HCMV, but not with Herpes simplex virus 1 (HSV-1) (Figure 1a). In addition, the ectopic expression of CD147-WT, but not the extracellular domain (residues 1–185) or intracellular domain (residues 230–269) deletion mutants of CD147 (CD147-dECD and CD147-dICD), restored promoter activity that was impaired by CD147-specific RNAi. These results confirm the specific contribution of CD147 to the induction of NF-κB and IFN-β by HCMV infection (Figure 1b). We next examined whether the endogenous CD147 was required for HCMV-induced antiviral signaling under physiological conditions. We found that the knockdown of CD147 expression in HFF cells decreased the activation of *IFNB1* and interferon-stimulated *ISG15* gene expression induced by HCMV infection, as well as expression of the NF-κB downstream genes *IL-6* and *TNF-α* (Figure 1c). Together, these data suggest that CD147 might block the HCMV-triggered early antiviral immune responses in a virus-specific manner.

### 3.2. CD147-Mediated HCMV-Triggered Antiviral Signaling via sCyPA-CD147 Interaction Activated ERK/NF-κB Pathway

**Figure 2:**
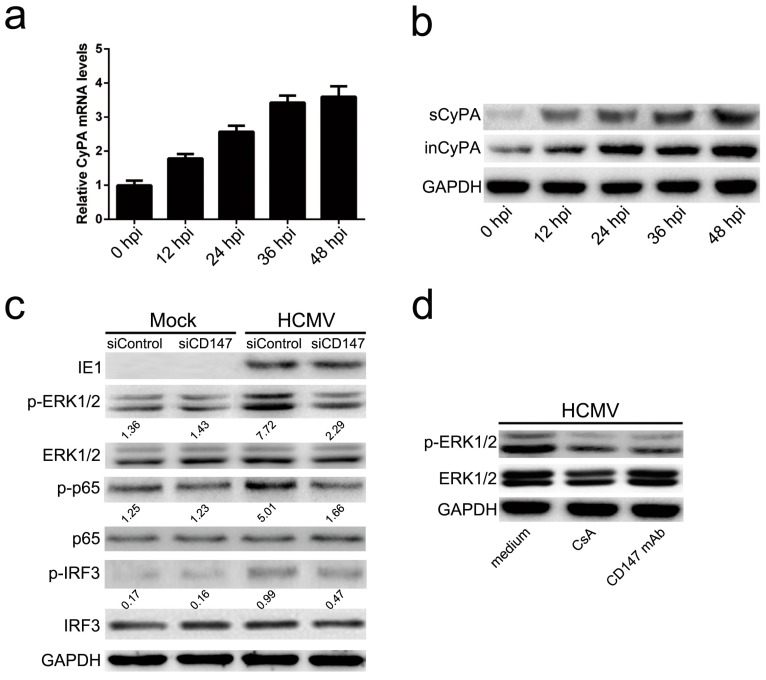
CD147 mediated HCMV-triggered antiviral signaling via the sCyPA-CD147 interaction activated ERK/NF-κB pathway. (**a**) Quantitative RT-PCR analysis of Cyclophilin A (CyPA) mRNA expression kinetics in HFF cells at different time points post-HCMV infection (MOI = 5); (**b**) Western blot analysis of secreted CypA (sCypA) protein levels and intracellular CypA (inCypA) in total protein lysates in culture mediums of HFF cells at the indicated times post HCMV infection (MOI = 5); (**c**) Western blot analysis of the phosphorylation levels of ERK1/2 (p-ERK1/2), NF-κB p65 subunit (p-p65) and IRF3 (p-IRF3) in response to HCMV infection for 6 h (MOI = 0.5) compared to mock infection (Mock) with the medium only. The intensities of bands were quantified by Image Lab 4.0 (Bio-Rad) and relative values normalized to GAPDH are indicated by the numbers under the lanes; (**d**) Analysis of the phosphorylation activation of ERK1/2, induced by HCMV infection for 6 h (MOI = 0.5) with the culture medium, pre-supplemented with cyclosporine (CsA) (5 μg/mL) or CD147 mAb (50 μg/mL).

From the results above, one might assume that CD147 acts as a receptor for HCMV infection. However, CD147 had no significant effect on HCMV entry, either when tested with a function-blocking antibody against CD147 to inhibit HCMV infection of HFF cells, or when the expression of CD147 was knocked down in U251 cells (Figure S2). In our previous report that agreed well with the findings of other studies, we found that CyPA can bind directly to the ectodomain of CD147, and could therefore be responsible for the pro-inflammatory effect of CD147 [18]. Thus, we had reason to speculate that HCMV infection might promote the expression and release of cellular CyPA, which could then lead to the activation of innate immune signaling through the interaction with CD147. To test this hypothesis, we analyzed the kinetics of CypA RNA expression by quantitative real-time PCR, intracellular CypA (inCypA), and secreted CypA (sCypA) protein levels by Western blot analysis following HCMV infection of HFF cells. As shown in Figure 2a, the relative mRNA levels of CypA RNA expression were gradually up-regulated after HCMV infection. Additionally, both inCypA and sCypA were highly expressed at 12 post-infection (hpi), followed by a gradual increase in expression level with prolonged HCMV infection (Figure 2b).

In previous studies, CyPA has been demonstrated to activate NF-κB by the ERK1/2 pathway through the CyPA-CD147 interaction, which promotes an inflammatory effect in monocytes and macrophages [28]. The transcription factors p65 and IRF3 play key roles in the induction of cellular antiviral responses. Phosphorylation of p65 and IRF3 is required for their activity and constitutes a key checkpoint. Thus, we next determined whether CD147-mediates HCMV-triggered antiviral signaling by the activation of ERK in HFF cells. As shown in Figure 2c, the phosphorylation levels of ERK1/2 (p-ERK1/2), NF-κB p65 subunit (p-p65), and IRF3 (p-IRF3) were increased in response to HCMV infection when compared to mock infection with the medium only. However, when endogenous CD147 expression was knocked down by lentiviral-based transduction of CD147-specific short hairpin RNA (siCD147), HCMV-induced phosphorylation levels of ERK1/2 and p65 were dramatically reduced relative to the siControl group. The decreased activation of ERK1/2 and p65 phosphorylation were more obvious than the level of IRF3, potentially emphasizing the importance and leading role of ERK and NF-κB activation in signal transduction. Furthermore, CD147-blocking mAb and cyclosporine (CsA), a novel inhibitor of CyPA that can specifically block the direct binding of CyPA to CD147, also inhibited the HCMV-induced phosphorylation of ERK1/2 (Figure 2d). Together, these results suggest that HCMV infection triggers the secretion of CyPA, which then activates the phosphorylation of ERK and downstream NF-κB through interaction with CD147, which ultimately promotes cellular antiviral signaling pathways.

### 3.3. HCMV miR-US25-1-5p Targeted and Down-Regulated CD147

**Figure 3:**
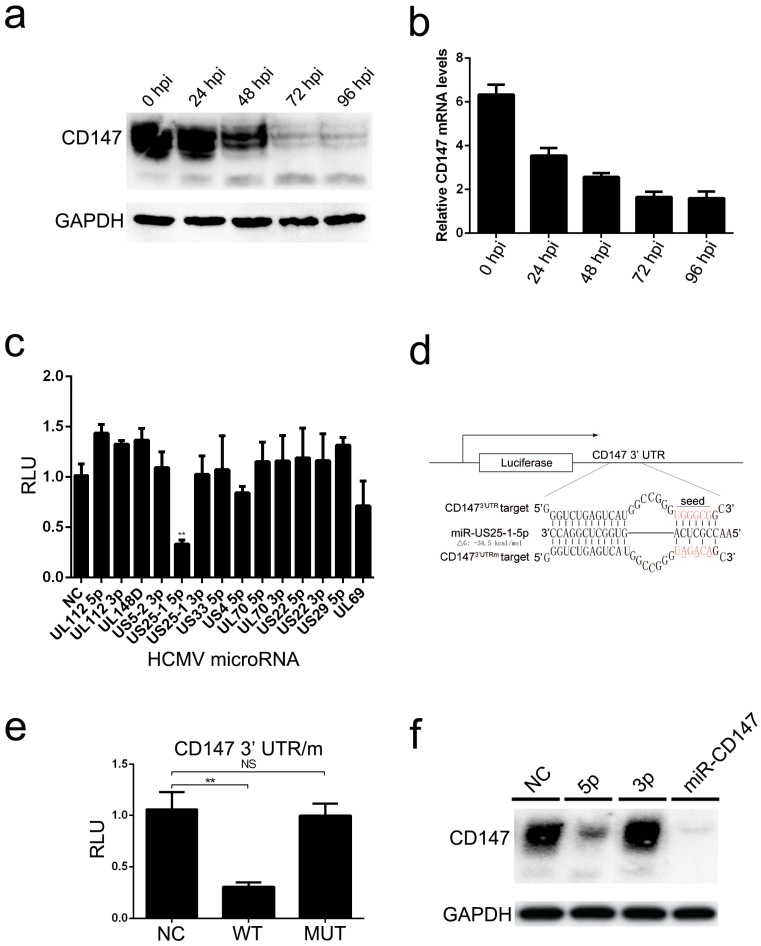
CD147 is targeted and down-regulated by HCMV miR-US25-1-5p. (**a**) Western blot analysis of CD147 protein levels in total protein lysates of HFF cells at the indicated times post HCMV infection (MOI = 5); (**b**) Quantitative RT-PCR analysis of CD147 mRNA expression levels in HFF cells at the corresponding time points post-HCMV infection (MOI = 5); (**c**) Dual luciferase reporter assay in 293 cells suggests that CD147 3′ UTR is targeted by miR-US25-1-5p. A luciferase reporter plasmid pmirGLO-CD147^3′UTR^ was tested; the 14 putative HCMV mimics are outlined in the abbreviations. Stars indicate potential down-regulated relative luciferase activities and the corresponding *p* value; (**d**) Schematic of miR-US25-1-5p-binding sites on CD147 3′ UTR (CD147^3′UTR^) and its mutant CD147^3′UTRm^, with the seed-recognizing site marked in red. The predicted hybrid free-energy of miR-US25-1-5p is indicated; (**e**) Confirmation of the predicted target site for miR-US25-1-5p. The 293T cells were co-transfected with miR-US25-1-5p mimic and the luciferase reporter pmirGLO-CD147^3′UTR^ (WT) or pmirGLO-CD147^3′UTRm^ (MUT) for 36 h before dual luciferase reporter assay; and, (**f**) Confirmation that miR-US25-1-5p (5p), but not miR-US25-1-3p (3p) specifically down-regulates endogenous CD147 in U251 cells transfected as indicated and harvested two days after transfection for Western Blot analysis. Commercial non-targeting control siRNAs (NC) and CD147-specific siRNAs (siCD147) were used for comparisons. The graphical data are presented as the means ± the SDs (*n* = 3); NS denotes not significant (*p* > 0.05); ** denotes *p* < 0.01.

Due to the potential role that CD147 may play in the HCMV early antiviral signaling mentioned above, we next investigated the influence of HCMV infection on the expression kinetics of cellular CD147 during different phases of the HCMV replication cycle. The protein expression levels of CD147 were dramatically down-regulated after 24 hpi of HCMV infection (Figure 3a), corresponding to a decrease in the relative RNA expression (Figure 3b). It is well known that microRNAs encoded by HCMV play essential roles in all aspects of the HCMV life cycle through the manipulation of many cellular signaling pathways. Thus, we next investigated whether the expression of CD147 was targeted and down-regulated by one of the HCMV-encoded 24 mature miRNAs previously identified in the reactome database (www.reactome.org). Using RNAhybrid (http://bibiserv.techfak.uni-bielefeld.de/rnahybrid/submission), we screened 14 HCMV-encoded mature miRNA candidates that might target the 3′ UTR region of CD147. To experimentally determine which HCMV-encoded miRNAs targets CD147, a pmirGLO dual luciferase reporter plasmid containing the 3′ UTR sequence of human CD147 downstream of the firefly luciferase gene was generated. Transfection of Human Embryonic Kidney 293 cells (293 cells) with each of the individual 14 putative HCMV miRNA constructs, and subsequent luciferase assay, suggested that CD147 was most likely targeted by miR-US25-1-5p (Figure 3c). The most likely target site for miR-UL25-1-5p was identified by RNAhybrid in the 3′ UTR region of CD147 mRNA (Figure 3d).

We next performed site-directed mutagenesis on the seed sequence of the predicted target site in the pmirGLO-CD147^3′UTR^ reporter construct. As indicated by Luciferase assays, the mutation of this site almost completely rescued the knockdown of CD147 by miR-UL25-1-5p in the wild type (WT) construct (Figure 3e). We then tested whether transfection of the miR-UL25-1-5p mimic could target endogenous CD147 in HCMV permissive U251 cells. When compared to cells that were transfected with a non-targeting control siRNA (NC) or miR-UL25-1-3p mimic, the CD147 protein level was appreciably reduced after the transfection of the miR-UL25-1-5p mimic. Notably, the efficiency was almost comparable to the commercial siRNA against CD147 (miR-CD147) (Figure 3f). These results indicate that miR-UL25-1-5p efficiently targets and down-regulates endogenous CD147 via a specific location site in the 3′ UTR sequence.

### 3.4. miR-US25-1-5p Inhibits CD147-Mediated Early Innate Immune Response to HCMV

**Figure 4:**
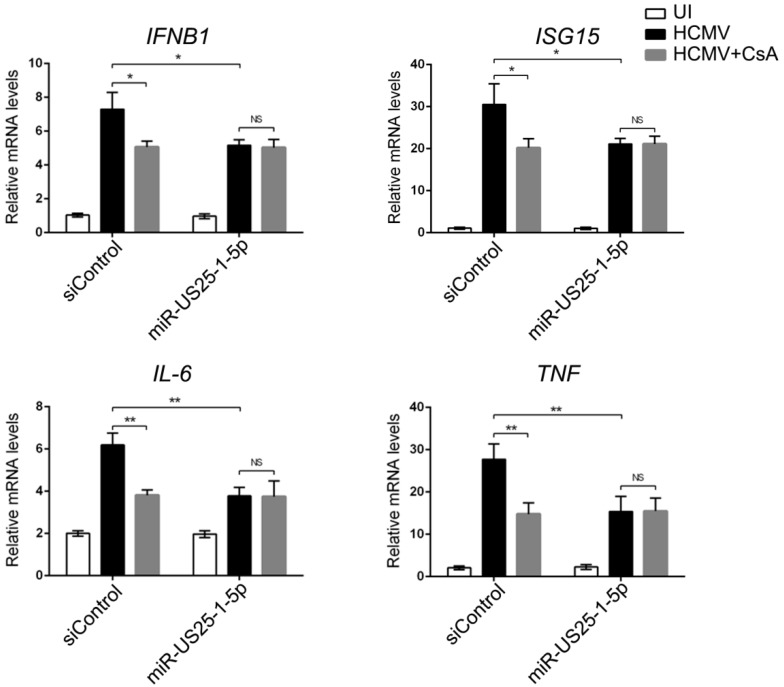
miR-US25-1-5p inhibits CD147-mediated early innate immune response to HCMV. Quantitative RT-PCR analysis of *IFNB1*, *ISG15*, *IL-6*, and *TNF* mRNA in siControl or miR-US25-1-5p stable HFF cells with HCMV uninfected (UI) or infected (MOI = 0.5) for 6 h and CsA present (HCMV + CsA) or absent in the culture medium. The graphical data are presented as the means ± the SDs (*n* = 3); NS denotes not significant (*p* > 0.05); * denotes *p* < 0.05, ** denotes *p* < 0.01.

We next determined if miR-US25-1-5p had the same effect as the CD147-specific short hairpin RNA on the CD147-mediated early innate immune response to HCMV. miR-US25-1-5p stably transfected HFF cells were constructed using the lentiviral-based transduction method. As shown in Figure S1b, the expression of CD147 obviously decreased in miR-US25-1-5p transduced cells when compared with the level in siControl cells that was observed by Western blot analysis. We then confirmed that knockdown of the CD147 expression by miR-US25-1-5p could specifically reduce the activation of *IFNB1* and interferon-related gene *ISG15* and the NF-κB downstream genes *IL-6* and *TNF-α* induced by HCMV early infection. These effects were similar to the effect of the CD147-specific siRNA studied above (Figure 4). More importantly, activation of these innate immune related genes by HCMV infection was also dramatically impaired in the siControl group when the culture medium was treated with cyclosporin A (CsA), a CyPA specific inhibitor that has been proven to block the CypA-CD147 interaction [29]. miR-US25-1-5p and CsA appeared to have redundant roles in the inhibition of the CD147-mediated early innate immune response to HCMV infection, since the addition of CsA to the miR-US25-1-5p transduced cell group had no effect on the transcriptional up-regulation of these genes. Taken together, these findings suggest that miR-US25-1-5p could function as a CD147-specific short hairpin RNA to inhibit the CD147-mediated early innate immune response to HCMV infection, and further supports the importance of the sCyPA-CD147 interaction in this process.

### 3.5. Mutation of miR-US25-1-5p or Ectopic Expression of CD147 Delays HCMV Lytic Propagation at Low MOI

**Figure 5:**
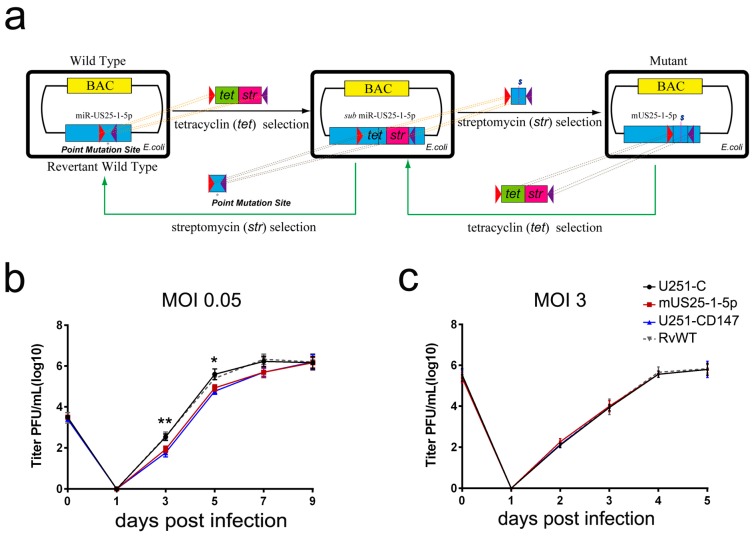
Mutation of miR-US25-1-5p or ectopic expression of CD147 can delay HCMV lytic propagation at low MOI. (**a**) Schematic construction of HCMV mUS25-1-5p and the revertant wild type (RvWT) with bacterial artificial chromosome (BAC) mutagenesis technique. U251 control cells (U251-C) were infected with the HCMV wild-type virus, RvWT or HCMV-mUS25-1-5p mutant virus (mUS25-1-5p) and CD147-overexpressing cells (U251-CD147) were infected with HCMV wild-type virus at an MOI of (**b**) 0.05 or (**c**) 3. Cell-associated and supernatant virus were harvested at the dpi indicated. Titers were determined by plaque assay. The values obtained were averages ± SD from triplicate experiments. * Denotes *p* < 0.05, ** denotes *p* < 0.01.

To further evaluate the biological significance of HCMV miR-US25-1-5p targeting of CD147, we generated a mutant HCMV (mUS25-1-5p) by HCMV BAC mutagenesis. To prevent any potential artifacts resulting from full sequence deletion, the mutants were constructed, by substituting only three nucleotides of the seed sequence of miR-US25-1-5p, corresponding to the putative CD147 mRNA 3′ UTR target site (Figure 5a). A revertant virus (RvWT) was constructed as a control. In addition, we constructed a U251 cell line that stably overexpresses CD147, as well as control U251 cell line. Effective loss of function in the interference of CD147 expression after infection with HCMV-mUS25-1-5p (Figure S3a), rescued down-regulation of CD147 with the RvWT (Figure S3b), and sustained overexpression of CD147 in pcDNA3.1-CD147 stably-transfected U251 cells for at least 14 days in comparison to control cells (Figure S1a), were confirmed by Western blot analysis.

Next, we examined the effect of HCMV-mUS25-1-5p and ectopic expression of CD147, relative to HCMV-WT (wild type), on different phases of the HCMV replication cycle. Multistep growth and single-step growth curves were analyzed in the constructed U251 cells that were infected with HCMV-mUS25-1-5p, RvWT, or HCMV-WT, at a low MOI of 0.05 or at a high MOI of 3, respectively. The supernatant and cell-associated virus were harvested together at the dpi indicated. HCMV titers were determined by virus plaque assay in HFF cells. As seen in Figure 5b, at a low MOI of 0.05, when multiple replication cycles of infection were measured, both HCMV-mUS25-1-5p in control U251 cells, and HCMV-WT in CD147 overexpressing U251 cells, displayed a similar slight delay in viral growth with statistical significance, but ultimately reached the same titer plateau as HCMV-WT in empty vector stably-transfected U251 cells. HCMV wild-type and the revertant RvWT showed similar growth kinetics, and reached the replication plateau at almost seven days post-infection (dpi) in U251-C cells. However, at the higher MOI, no differences in virus yield were observed, either in cells infected with the HCMV mutant virus miR-US25-1-5p, or in cells overexpressing CD147 (Figure 5c). Similar results were observed when the mutant HCMV-mUS25-1-5p and wild type viruses were used to infect HFF cells. These results indicate that HCMV miR-US25-1-5p targeting of CD147 might promote the course of HCMV Lytic propagation at low MOI.

## 4. Discussion

Numerous cell surface glycoproteins, such as β2 microglobulin, annexin II, CD13, platelet-derived growth factor receptor-α (PDGFR-α), epithelial growth factor receptor (EGFR), and β1 integrins, have been suggested to act as receptors facilitating HCMV entry [30]. However, none of the suggested glycoproteins has been validated as a bona fide receptor that is necessary for HCMV infection of all the susceptible cell types. The in vivo broad tissue tropism of HCMV infection and differences in permissiveness for different cell types suggest that HCMV likely uses distinct cell surface receptors depending on the target cells. Thus, identifying the specific HCMV cellular receptors is important for understanding the pathogenesis of HCMV. CD147/EMMPRIN is a transmembrane receptor glycoprotein that is belonging to the immunoglobulin superfamily and expressed at varying levels in many tissue cells. Notably, several bacterial pathogens, such as Neisseria meningitidis and Plasmodium falciparum, as well as many viruses, including HIV-1, HBV, SARS-CoV, and measles virus (Mev), have been previously found to infect target cells via CD147, indicating that it might constitute an evolutionarily conserved pathogen receptor for infection and spread within tissues and organisms [31]. Unfortunately, neither the infection-blocking experiment using CD147-specfic antibody, nor the viral infection test with CD147-knockdown cells, showed any difference in facilitating HCMV entry (Figure S2). Since a low passage clinical HCMV strain NR-1 was used in these experiments, we assumed that CD147 does not function as a broad-spectrum receptor for HCMV infection, at least in typical in vitro permissive cell lines.

**Figure 6:**
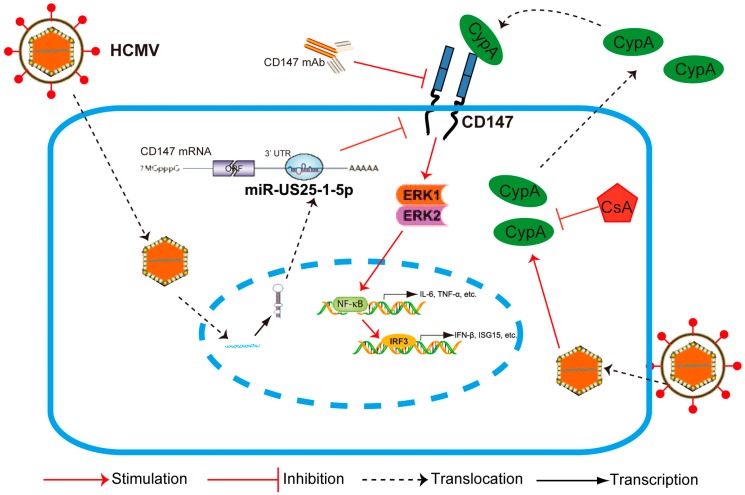
General model for HCMV miR-US25-1-5p in attenuating CD147/EMMPRIN-mediated HCMV-triggered early antiviral response. Upon HCMV infection, intracellular CypA is released. As a paracrine proinflammatory cytokine, CyPA interacts with CD147 of those uninfected cells and tissues, and stimulates the phosphorylation of ERK1/2. The activation of NF-κB then occurs, followed by a subsequent activation of the IRF3 phosphorylation and the orchestrated NF-κB-dependent, IRF3 plus NF-κB-dependent immune responses. HCMV-encoded miR-US25-1-5p targeting CD147, CD147-blocking mAb, and an inhibitor of CyPA (CsA) can specifically inhibit this process.

In addition to the pathogen recognition receptor function, most transmembrane receptor proteins have been proposed to mediate signal transduction pathways immediately upon pathogenic particle attachment and penetration. CD147 belongs to the Type I membrane proteins and as a candidate tumor biomarker, its expression is up-regulated in breast cancer tissue, lung carcinoma tissue, and bladder cancer tissue, along with a tumor’s malignance, invasiveness, and metastasis [32]. CD147 has been extensively studied since the discovery of its function in tumor progression and metastasis. Here, we have explored a previously unknown role for CD147 in the host innate immune defense against HCMV invasion. Cells detect HCMV infection as early as four to eight hours post-infection (hpi), responding by producing NF-κB-dependent cytokines and an early peak of antiviral IFN [5,33]. Our experiments indicate that endogenous CD147 is required for the maximum activation of NF-κB and IFN-β-associated antiviral signaling that is triggered by HCMV infection, possibly having a redundant effect with other cell surface receptor glycoproteins (Figure 1). Many tumor-related proteins, such as Programmed death 1 (PD-1) and PTEN, are known to play key roles in regulating the innate immunity against viral and bacterial infection [34,35]. However, the involvement of CD147 in early antiviral immunity appears to be specific to HCMV infection, since it does not extend to other viruses, like HSV-1. Since the signaling pathways of CD147 remain to be well defined, we next demonstrated that the phosphorylation of ERK1/2 and then nuclear translocation of NF-κB might play a leading role in CD147-mediated HCMV-triggered antiviral signal transduction, with a subsequent activation of the IRF3 phosphorylation and the orchestrated NF-κB-dependent cytokines, IRF3 plus NF-κB-dependent interferon responses (Figure 6). This finding can be supported by the fact that NF-κB is a key transcriptional enhancesome-component of the IFN-β, according to previous research [36,37].

Research has increasingly suggested that the modulation of the host immune microenvironment is not only important for tumor virus-induced inflammatory carcinogenesis, but it is also critical for persistent and latent viral infection. For example, cmvIL-10, a viral mimic of human IL-10 acquired from the cellular genome, binds with a high affinity to the human IL-10 receptor, and limits the innate and adaptive immune responses even more efficiently than IL-10 itself [38]. In this study, we have demonstrated that HCMV infection leads to the expression and secretion of a cellular chemokine, CyPA. Studies show that CypA, as an extracellular ligand for its receptor CD147, can be secreted by various cell types, including vascular smooth muscle cells, macrophages, and fibroblasts, and triggers a cascade of inflammatory responses [39]. We demonstrated that CD147-mediated HCMV-triggered phosphorylation of ERK1/2, and the activation of NF-κB depends on the release of CyPA from HCMV infected cells and its interaction with CD147 (Figure 2). Although HCMV is highly species-specific with no animal model yet known to support its replication, we speculate that secreted extracellular CypA, triggered by HCMV infection, acts through a paracrine mechanism, to stimulate uninfected cells to defend against a second wave of infection by the progeny virus through the interaction with CD147.

Moreover, we found that endogenous CD147 was targeted by HCMV-encoded miR-US25-1-5p at the 3′ UTR, which could replace CD147-specific siRNA mimics in inhibiting the CD147-mediated early innate immune response to HCMV infection (Figure 4). Mutation of the HCMV miR-US25-1-5p seed sequence or ectopic expression of CD147 resulted in delayed multistep growth curves at low MOI, suggesting that miR-US25-1-5p targeting of CD147 is biologically significant (Figure 5b). MicroRNAs (miRNAs) are small noncoding RNA molecules that generally target 3′ untranslated regions (3′ UTR) of the mRNAs. Since the discovery of HCMV-encoded miRNAs, at least 26 mature miRNA species encoded by 14 pre-miRNAs have been identified and reported to regulate multiple aspects of viral and cellular processes, including viral replication, immune evasion, formation of the virion, and eukaryotic translation [40]. The pre-HCMV-miR-US25-1, from which the mature miR-US25-1-5p is derived, is encoded by the HCMV US24 and US26 intergenic regions [41]. miR-US25-1-5p has been reported to be transcribed upon infection and highly expressed at 24 hpi, followed by a gradually increased expression, during the course of HCMV infection [42]. One might expect that the cells would respond differently to infection with the mutant virus (HCMV-mUS25-1-5p), at late times of HCMV infection, when the expression levels of CD147 were obviously down-regulated by miR-US25-1-5p. However, we found no difference in the expression of ISGs after infection with the mutant virus. Based on previous studies and our overall findings, the reasons might be complex. At the late stage of infection, HCMV often uses multi-faceted and redundant strategies to regulate immune responses. Thus, the effect of natural miR-US25-1-5p may not be able to be detectable at late times of HCMV infection. We speculate that secreted extracellular CypA, which is triggered by HCMV infection, acts primarily to defend uninfected cells against progeny HCMV virus released from neighboring infected cells. As a result, the observed lack of differences in virus yield at the higher MOI, irrespective of the mutation of miR-US25-1-5p in the targeting of CD147 mRNA 3′ UTR or ectopic expression of CD147, are understandable (Figure 5c).

In summary, we have provided the first evidence that CD147, a non-HCMV receptor glycoprotein, is targeted by HCMV-encoded miR-US25-1-5p. This may be a tactic that is acquired by HCMV to antagonize the early innate immune response to benefit HCMV chronic infection. Additionally, HCMV-induced paracrine effects of the HCMV-induced expression of CypA could cause an immune microenvironment called “smoldering inflammation”, which could potentially contribute to the development of many HCMV inflammatory disorders.

## Supplementary Materials

The following are available online at www.mdpi.com/1999-4915/9/12/365/s1.
