# Supplementary figures and images for: Human Cytomegalovirus Encoded miR-US25-1-5p Attenuates CD147/EMMPRIN-Mediated Early Antiviral Response
Source: Viruses. 2017 Dec 1;9(12):365. doi: 10.3390/v9120365 (PMC5744140; doi:10.3390/v9120365)

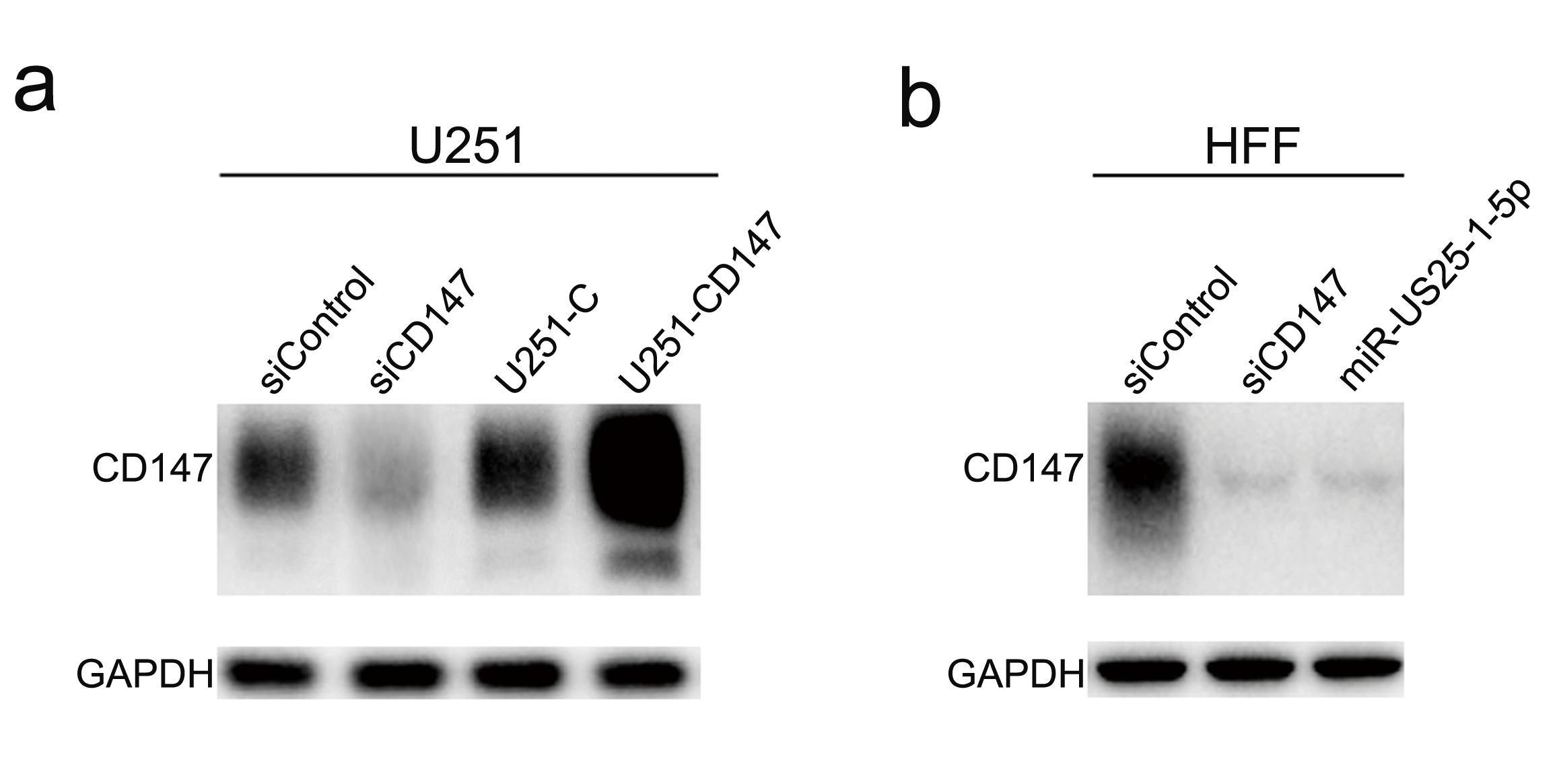

Supplement: Supplementary file 1 [file viruses-09-00365-s001.zip › Supplementary figures/Fig 1S.tif]

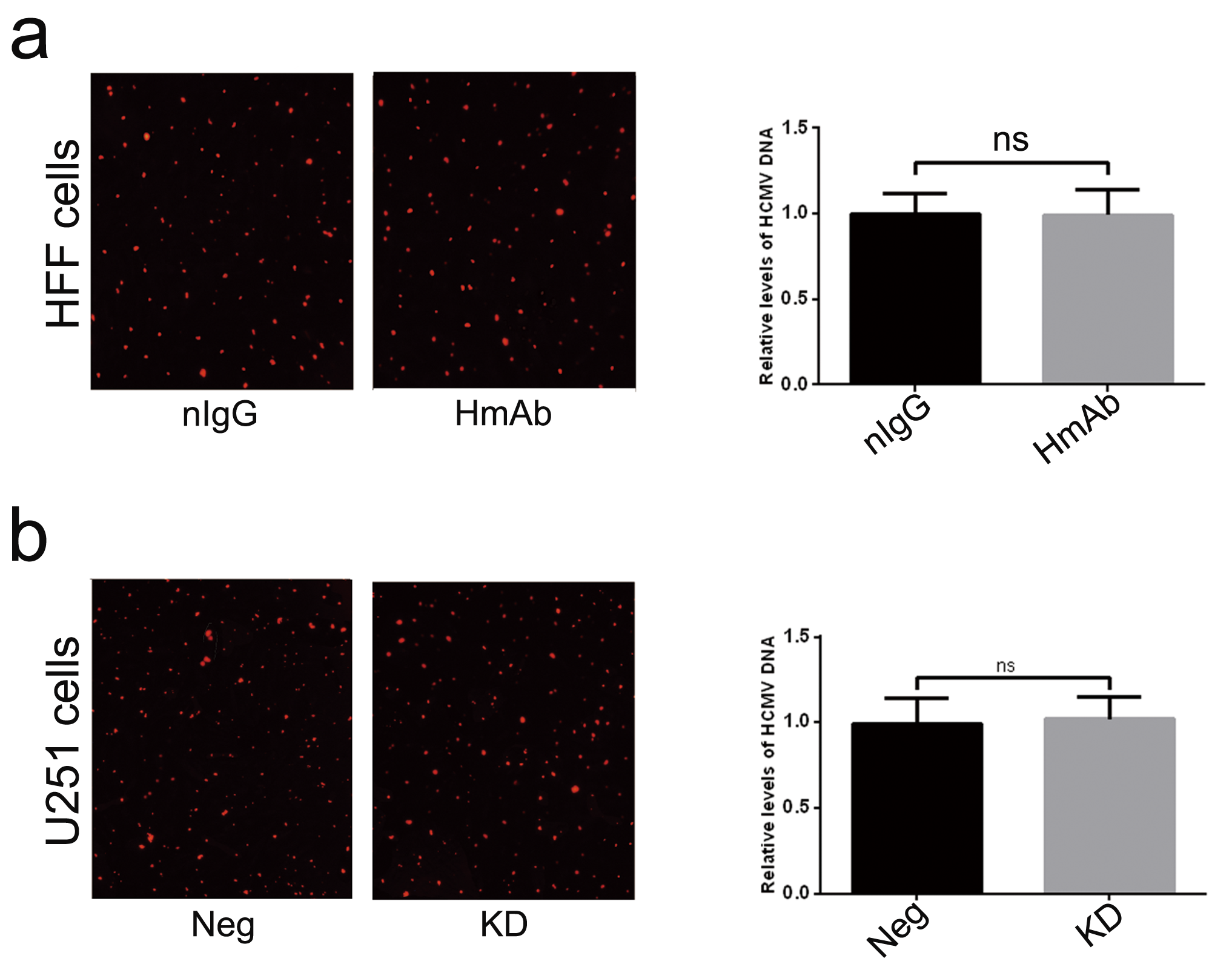

Supplement: Supplementary file 1 [file viruses-09-00365-s001.zip › Supplementary figures/Fig 2S.tif]

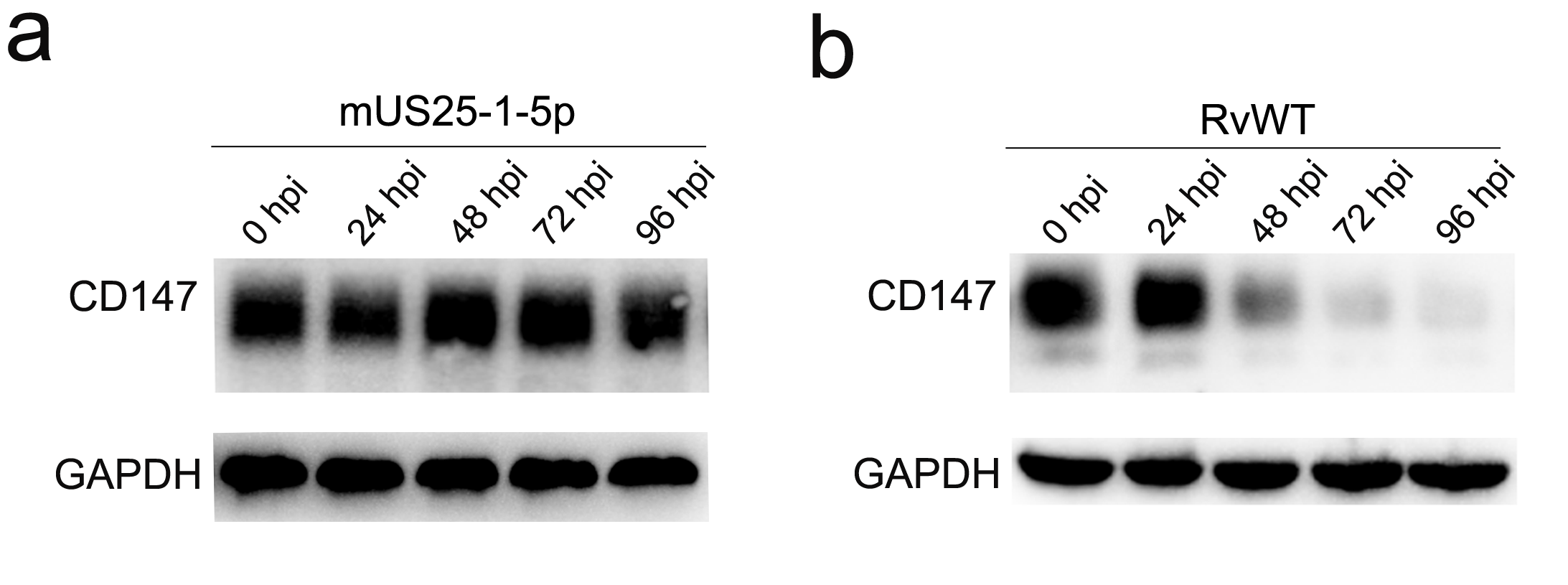

Supplement: Supplementary file 1 [file viruses-09-00365-s001.zip › Supplementary figures/Fig 3S.tif]
